# Lung Ultrasound Role in Diagnosis of Neonatal Respiratory Disorders: A Prospective Cross-Sectional Study

**DOI:** 10.3390/children10010173

**Published:** 2023-01-16

**Authors:** Rania Ismail, Nehal M. El Raggal, Laila A. Hegazy, Hossam M. Sakr, Osama A. Eldafrawy, Yasmin A. Farid

**Affiliations:** 1 Pediatric Department, Faculty of Medicine, Ain Shams University, Cairo 11566, Egypt; 2 Radiology Department, Faculty of Medicine, Ain Shams University, Cairo 11566, Egypt; 3 Children Hospital, Ain Shams University, Cairo 11566, Egypt

**Keywords:** chest X-ray, lung ultrasound, neonatal respiratory disorders, respiratory distress

## Abstract

Lung ultrasound (LUS) has become one of the most exciting applications in neonatal point-of-care ultrasound (POCUS), yet still lacks routine clinical use. This study assesses the utility of LUS for neonatal respiratory disorders (NRDs) diagnosis and follow-up compared to chest X-ray (CXR). A prospective cross-sectional study was conducted on 100 neonates having NRDs with a gestational age ≥28 weeks, excluding those having multiple congenital anomalies, chromosomal aberrations, hydrops fetalis and/or heart failure. CXR and LUS were done on admission for diagnosis and were repeated after 7 days, or if needed earlier within the 7 days. The diagnosis of NRDs by CXR and LUS on admission and after 7 days was comparable (*p* > 0.05). LUS diagnosis sensitivity and specificity for respiratory distress syndrome, pneumonia, meconium aspiration syndrome, pneumothorax and pulmonary atelectasis were 94.7/100%, 97.5/95%, 92.3/100%, 90.9/98.9% and 100/97.8%, respectively. The total agreement between LUS and CXR was 98.5% with 95% CI (0.88 to 0.92). LUS and CXR had considerable agreement in the diagnosis of NRDs. Being a reliable bedside modality of diagnosis and safer than CXR, LUS may be considered an alternative method for the diagnosis of neonates with NRDs.

## 1. Introduction

Respiratory diseases in neonates are the most common indication for admittance to the neonatal intensive care unit (NICU). Moreover, they are the most frequent cause of morbidity and early mortality (0–7 days of age) in neonates [1,2].

Clinical signs and plain chest X-ray (CXR) are the basics of neonatal respiratory disease diagnosis, which usually causes a diagnostic dilemma for clinicians due to the poor sensitivity and specificity of the signs and symptoms, and chest X-ray often does not solve this dilemma. Hence, this may lead to retarded or inappropriate management due to imprecise diagnosis [3,4].

Lung ultrasound (LUS) examination over the past decade as an alternative to CXR for the diagnosis of neonatal lung diseases has been explored in several works. In neonates, LUS imaging can be very useful, with the advantage of neonatal anatomical features having small thoracic width and lung mass with a thin chest wall. This allows a satisfactory visualization of the lungs, though still indirect [4].

LUS imaging is of particular benefit as it is a radiation-free method; its application is relatively easily learned, and it is less technically demanding than other sonographic examinations [5].

The European Society of Pediatric and Neonatal Intensive Care (ESPNIC) guidelines on point-of-care ultrasound (POCUS) usage in neonates and children clearly highlight the importance of LUS in the evaluation of the critically ill patient. [6].

The aim of this study was to assess the utility of LUS for the diagnosis of NRDs compared to CXR.

## 2. Materials and Methods

This prospective cross-sectional study was carried out from October 2017 to April 2019, enrolling 100 neonates admitted to the neonatal intensive care unit (NICU) of the Children’s Hospital of Ain Shams University, Cairo, Egypt. Inclusion criteria were neonates with a gestational age ≥28 weeks who were suffering from respiratory distress disorders; exclusion criteria were neonates having multiple congenital anomalies, chromosomal aberrations, hydrops fetalis and/or heart failure. Expert neonatologists managed enrolled neonates according to the NICU protocol [7].

Downes and Silverman–Andersen clinical scores were applied to evaluate neonatal respiratory distress severity. The Silverman–Anderson score was ideally used for preterm infants, and the Downes score was used for term infants [8,9,10].

Plain CXR and LUS were done on admission for diagnosis and were repeated after 7 days, or if needed earlier within the 7 days, by the treating neonatologist in parallel to the clinical assessment and laboratory findings to diagnose the cause of respiratory distress. CXR images were posterior–anterior view, using the digital GE (General Electric) Optima XR220 AMX pro series X-ray machine, (GE HealthCare, Chicago, IL, USA).

CXR findings were interpreted and used as the gold standard to diagnose and differentiate variable etiologies of neonatal respiratory distress: transient tachypnea of the newborn (TTN), respiratory distress syndrome (RDS), neonatal pneumonia, meconium aspiration syndrome (MAS), pulmonary interstitial emphysema (PIE), pneumothorax (PTX), pleural effusion (PE), pulmonary atelectasis (PA) and congenital diaphragmatic hernia (CDH) [1,2,11,12,13,14,15,16].

Lung ultrasound (LUS) examination was done using the GE Logiq 400 pro series ultrasound machine, with a linear 8 MHz microprobe, (GE HealthCare, Chicago, IL, USA). LUS was performed by a single radiology consultant. The neonatologists were blind to lung ultrasound diagnoses. Neonates were examined lying in a supine position and in a resting state with the surrounding light intensity kept constant and low; as the phototherapy device was turned off if it was on. Gentle handling, including quiet voice tones and fine touching, was applied to avoid stressful situations or cause for crying. In order to pacify babies on continuous positive airway pressure (CPAP) or nasal cannula, oral dextrose drops or a pacifier were given, and stimulation of non-nutritional sucking was applied, while others on ventilation might have been sedated by intravenous Dormicum.

During the study, LUS examination was done using the following LUS score: every lung was divided into 3 areas (upper anterior, lower anterior and lateral) and a linear microprobe was used in lung examination through both transverse and longitudinal scans. For every lung area, a point score from 0 to 3 was applied (total score varying from 0 to 18). The LUS score was allocated as follows:

0: Denotes A-pattern (defined by the existence of the A-lines only, which emerges from the pleural line reverberation artifact);

1: B-pattern (defined by the existence of ≥3 well-spaced B-lines; B-lines are lines reaching the screen edge in the absence of fading);

2: Severe B-pattern (defined by the existence of coalescent and crowded B-lines with or without consolidations restricted to the subpleural space);

3: Extended consolidations.

A-lines denote pleural reflection because of ultrasound diffusing through an air-filled lung; B-lines denote fluid filling the interstitium (and the alveolar space if they become coalescent). LUS diagnostic criteria for neonatal respiratory diseases were according to Corsini et al. [17,18].

### 2.1. Sample Size Calculation

The Epi Info program was applied for the sample size of 100 neonates. Calculation was guided by the following data: power of the test = 80%; confidence level = 95%; accepted margin of error = 5%. This was a prospective cross-sectional study, with a risk ratio of 3, total sample: 100 neonates, patients selected by randomization.

### 2.2. Statistical Analysis

Data were analyzed using IBM© SPSS© Statistics version 22 (IBM© Corp., Armonk, NY, USA) and MedCalc© version 14 (MedCalc© Software bvba, Ostend, Belgium). Categorical variables were presented as number (%). Nominal data were compared using Pearson’s chi-squared test or Fisher’s exact test, when appropriate. The chi-squared test for linear-by-linear association was applied to compare ordinal data. A two-sided *p*-value < 0.05 was considered statistically significant. In the differential diagnosis of RD, concordance between LUS and CXR was evaluated with the Cohen unweighted κ statistic. LUS sensitivity and specificity for every disease were calculated using CXR as a gold-standard tool for diagnosis.

## 3. Results

One hundred neonates were enrolled; fifty-five were full-term neonates (55%), twenty-two were late preterm neonates with a gestational age of 34–36 weeks (22%) and twenty-three were preterm neonates with a gestational age of less than 34 weeks (23%). The 100 neonates had 100 initial diagnoses based on clinical and CXR examinations. Twenty-one of them developed multiple diagnoses during the first week of admission, resulting in a total of 122 diagnoses by CXR and 125 diagnoses by LUS. The neonatal clinical and demographic data are presented in Table 1. Surfactant therapy was given to 18 neonates with RDS and 8 neonates with MAS.

Diagnoses obtained by CXR and LUS on admission were comparable (*p* = (>0.05)). However, diagnosis of RDS and MAS by CXR was higher than LUS, yet statistically insignificant; 19 neonates were diagnosed as RDS by CXR versus 18 neonates by LUS (*p* = (0.856)), and 13 neonates were diagnosed as MAS by CXR versus 12 neonates by LUS (*p* = (0.830)). Nevertheless, pneumonia was diagnosed more by LUS, but with a statistically insignificant difference; 39 neonates were diagnosed with pneumonia by CXR versus 41 neonates by LUS (*p* = (0.773)). Moreover, CXR and LUS diagnoses after 7 days were comparable (*p* = (>0.05)), and diagnoses were equivalent to those on admission. In addition to neonates who developed new diagnoses, pleural effusion (1 neonate) and pneumothorax (11 neonates) were diagnosed by both CXR and LUS, with *p* = (1.000), and as for atelectasis, 10 neonates were diagnosed by CXR versus 12 neonates diagnosed by LUS (*p* = (0.652)).

Regarding the different sonographic findings of different respiratory distress disorders in the studied neonates, double lung point (DLP) is a diagnostic criterion for TTN (100%) of neonates, and no consolidation was found in any case. Furthermore, lung point is a very specific finding for pneumothorax diagnosis, and was present in 100% of cases. Additionally, abdominal organs inside the chest were a specific finding in CDH (100% of cases). On the other hand, abnormalities of pleural lines were present in 100% of cases of RDS, TTN, MAS, pleural effusion and CDH, and in 76% of pneumonia cases, yet were not present in pneumothorax or pulmonary atelectasis. Moreover, the primary ultrasonic feature of RDS (84% of cases) was lung consolidation with air bronchograms without DLP. Consolidation was present in 100% of cases of RDS, pneumonia, MAS and pulmonary atelectasis.

LUS showed high accuracy in the diagnosis of different respiratory disorders among the studied neonates, as presented in Table 2.

The agreement and disagreement between LUS and CXR for each respiratory disease diagnosis for the studied neonates is presented in Table 3.

## 4. Discussion

In the present study, LUS was highly accurate in the diagnosis of the most frequent respiratory neonatal diseases (i.e., RDS, TTN, pneumonia, MAS, pneumothorax, CDH, pleural effusion and pulmonary atelectasis), with 98.5% total agreement with CXR. Regarding RDS diagnosis, LUS showed sensitivity and specificity of 94% and 100%, respectively, with 99% agreement with CXR. These results were similar to those of Corsini et al., as the sensitivity and specificity in their study were 94% and 100%, respectively [19].

Additionally, Liang et al. and Vergine et al. discovered that in RDS diagnosis LUS sensitivity and specificity were 95.6% and 94.4%, respectively, which were higher than those of CXR [1,20].

This study found that lung consolidation with air bronchograms, lung sliding reduction or disappearance and abnormalities of pleural line and white lung were LUS’s main RDS diagnostic features; this was in agreement with Shui et al. [21]. Moreover, Copetti et al. confirmed that the previously mentioned RDS features have 100% sensitivity and specificity for RDS diagnosis [22].

The transthoracic approach was applied for the studied neonates, as the transthoracic technique had better sensitivity and specificity than the trans-abdominal approach in RDS diagnosis. Owing to the transthoracic technique’s high specificity, the number of false-positive diagnoses, as well as unnecessary extra examinations or interventions, would be reduced [20].

Regarding pneumonia diagnosis, LUS had sensitivity and specificity of 97.5% and 95%, with 97% agreement with CXR. The 3% disagreement was a result of three false positive cases that were confirmed to be RDS, MAS and pneumothorax. Nevertheless, Corsini et al. confirmed 100% sensitivity and specificity and 100% agreement with CXR [18,19].

The LUS features for pneumonia in both children and neonates are similar, where air bronchograms, lung consolidation (100% of cases) and abnormalities of pleural line (76%), with or without pleural effusion, are the main features. In children, viral and bacterial pneumonia findings were clearly distinguished from each other by LUS during the H1N1 influenza pandemic in 2009 [23].

Moreover, LUS was used to evaluate neonates with SARS-CoV-2-positive PCRs in respiratory samples, with the following findings confirming the diagnosis: B-lines, consolidation and spared areas without pleural effusion nor pneumothorax were detected [19].

Correspondingly, LUS could be used in screening and diagnosing novel coronavirus disease-19 pneumonias (nCoV-19) instead of chest CT [24].

Regarding TTN diagnosis, we revealed 100% agreement between LUS and CXR with sensitivity and specificity of 100%, and this was comparable to the results of Corsini et al., who confirmed sensitivity and specificity of 100% and 98% [19].

Double lung point (DLP) was present in 100% of TTN cases in this study. DLP is a sharp increase in echogenicity in the lower lung fields of TTN neonates, and it is considered a diagnostic feature [25,26].

However, Corsini et al. showed that interstitial edema was the main ultrasonic imaging feature of TTN (represented by B-lines) in 100% of cases, and normal areas (represented by A-lines) [19].

Moreover, the current results found that the primary ultrasonic feature of RDS cases (84%) was lung consolidation with air bronchograms without DLP. Comparably, Jing et al. reported the same results, hence LUS could easily differentiate TTN from RDS [25].

As for MAS diagnosis by LUS, this study revealed sensitivity and specificity of 92.3% and 100%, respectively, and 99% agreement with CXR, where one case was misdiagnosed as pneumonia. This might agree with Corsini et al., who confirmed 100% agreement of LUS with CXR and 100% for both sensitivity and specificity, and Liu et al., who also confirmed LUS as a reliable method to diagnose MAS [19,27].

In a study by Piastra et al., they revealed that coalescent or sparse B-lines, consolidations, atelectasis and bronchograms were the LUS findings in all MAS cases (100%), with variable severity. These signs irregularly exist all over the lungs, and as the meconium-driven inflammation progresses over time they might change. Additionally, these LUS findings were similar to those of pneumonia, explaining our false-negative case [28].

Regarding pneumothorax diagnosis by LUS, this study outcome recorded sensitivity and specificity of 90.0% and 100%, respectively, and 98% agreement with CXR. This disagreement was due to one false-positive case, which was pneumonia, and one false-negative case, which was MAS. This might be close to the outcome of Corsini et al., who showed 80% sensitivity and 100% specificity with 98% agreement [19], and Lichtenstein et al., who showed 79% sensitivity and 100% specificity [29].

In adults, the CT scan is the gold standard for pneumothorax diagnosis [30]. In contrast, CT was less accurate than CXR and LUS in the diagnosis of neonatal pneumothorax, and LUS had the same reliability as CXR, attributed to the neonates’ anatomical features (smaller lung mass, thoracic wall and thin chest wall) [31].

Regarding CDH and pleural effusion diagnosis by LUS, this study showed sensitivity and specificity of 100% each, and these results were comparable to those of Corsini et al. [19].

Furthermore, for diagnosing neonatal pulmonary atelectasis by LUS, the current results showed sensitivity and specificity of 100% and 97.8%, respectively, with 98% agreement with CXR. These results were comparable with those of Liu et al. regarding sensitivity results, revealing a sensitivity of 100%; however, their specificity was 75% [32]. Moreover, Lichnestien et al. revealed 100% sensitivity for LUS in diagnosing lung atelectasis in children [33].

In another study, Acosta et al. investigated LUS accuracy in diagnosing anesthesia-induced atelectasis in children, utilizing lung magnetic resonance imaging (MRI) as a reference, where LUS showed 88% sensitivity and 89% specificity. This is due to the state of collapsed and non-aerated areas in the parenchyma of the lung because of parenchymal compression (non-obstructive atelectasis) or bronchial obstruction (obstructive atelectasis), which is characteristic of pulmonary atelectasis. Therefore, the main LUS features of atelectasis, present in 100% of cases, were lung consolidation and air bronchograms. A static air bronchogram was found in most of the cases, yet a dynamic air bronchogram was not present. Hence, the presence of a dynamic air bronchogram can exclude pulmonary atelectasis [34].

Consequently, the current results revealed 98.5% agreement between LUS and CXR, and this was consistent with Melet et al. as CXR was comparable to LUS in the diagnosis of neonatal respiratory diseases when they studied 74 patients by LUS; they diagnosed 25 neonates with RDS (33.78%), 13 neonates with pneumonia (17.56%), 25 neonates with TTN (33.7%), 9 neonates with MAS (12.16%) and 4neonates with pleural effusion (5.40%) [35]. In addition, Corsini et al. proved that the agreement between LUS and CXR diagnosis was 91% (122/134 diagnoses), and they allocated 9% disagreement between LUS and CXR due to six false-positive cases (one congenital pulmonary airway malformation and five cases with pleural effusions), two false-negative LUS diagnoses of pneumothorax and four different diagnoses [19].

The limitation of this study was the relatively small size of our population, which did not allow for a robust evaluation of the concordance between LUS and CXR for some uncommon conditions (i.e., congenital pulmonary airway malformation, pleural effusion and congenital diaphragmatic hernia).

## 5. Conclusions

LUS and CXR had considerable agreement in the diagnosis of NRDs. Being a reliable bedside modality of diagnosis and safer than CXR, LUS may be considered an alternative method for the diagnosis of neonates with NRDs.

## Figures and Tables

**Table 1:** Demographic and clinical data of the studied neonates.

	Studied Neonates (*n* = 100)
Gender: Male	57 (57%)
Mode of delivery: CS	90 (90%)
Gestational age: (weeks) M ± SD	36.3 ±2.816
Birth weight (g) M ± SD	2471.2 ±753.012
Respiratory support during CXR and LUS examination Invasive ventilation Noninvasive ventilation	36 (36%) 14 (14%)
Surfactant therapy	26 (26%)

CS: caesarean section. CXR: chest X-ray. LUS: lung ultrasound. M ± SD: mean and standard deviation.

**Table 2:** Sensitivity, specificity and accuracy regarding LUS in studied respiratory diseases.

Diagnosis (N)	TP	TN	FP	FN	Sensitivity	Specificity	Accuracy
RDS (19)	18	81	0	1	94.7%	100.0%	0.990
Pneumonia (39)	39	58	3	0	97.5%	95.0%	0.960
TTN (28)	28	72	0	0	100%	100.0%	1.000
MAS (13)	12	87	0	1	92.3%	100.0%	0.990
CDH (1)	1	99	0	0	100.0%	100.0%	1.000
PE (1)	1	99	0	0	100.0%	100.0%	1.000
PNX (11)	10	88	1	1	90.9%	98.9%	0.980
Atelectasis (10)	10	88	2	0	100.0%	97.8%	0.980

RDS: respiratory distress syndrome. TTN: transient tachypnea of newborn. MAS: meconium aspiration syndrome. N: number of patients. CDH: congenital diaphragmatic hernia. PE: pleural effusion. PNX: pneumothorax. TP: true positive. TN: true negative. FP: false positive. FN: false negative.

**Table 3:** Agreement between LUS and chest X-ray in the studied neonates.

	Agreement %	Disagreement %	Kappa Agreement	95% CI
RDS	99.0%	1.0%	0.967	0.902 to 1.000
Pneumonia	97.0%	3.0%	0.917	0.838 to 0.997
TTN	100.0%	0.0%	1.000	1.000 to 1.000
MAS	99.0%	1.0%	0.954	0.865 to 1.000
CDH	100.0%	0.0%	1.000	1.000 to 1.000
PE	100.0%	0.0%	1.000	1.000 to 1.000
PNX	98.0%	2.0%	0.898	0.758 to 1.000
Atelectasis	98.0%	2.0%	0.898	0.759 to 1.000
Total agreement	98.5%	1.5%	0.940	0.888 to 0.992

RDS: respiratory distress syndrome. TTN: transient tachypnea of newborn. MAS: meconium aspiration syndrome. CDH: congenital diaphragmatic hernia. PE: pleural effusion. PNX: pneumothorax. *kappa test*.

## Data Availability

The datasets generated or analyzed during the current study are available from the corresponding author upon reasonable request.
